# Primary Repair for Treating Acute Proximal Anterior Cruciate Ligament Tears: A Histological Analysis and Prospective Clinical Trial

**DOI:** 10.3389/fbioe.2022.913900

**Published:** 2022-05-27

**Authors:** Yue Yang, Zhuangzhuang Jin, Jianghua Luo, Delong Zhang, Peng Shen, Dianbin Zheng, Donghao Liu, Lunhao Bai

**Affiliations:** ^1^ Department of Orthopaedic Surgery, Shengjing Hospital of China Medical University, Shenyang, China; ^2^ Department of Emergency Medicine, Shengjing Hospital of China Medical University, Shenyang, China; ^3^ Department of Orthopedic Surgery, Zhongshan Hospital Affiliated to Xiamen University, Xiamen, China

**Keywords:** anterior cruciate ligament, acute proximal tears, primary repair, reconstruction surgery, histological study, prospective clinical trial

## Abstract

Reconstruction surgery for acute proximal anterior cruciate ligament (ACL) tears remains controversial. Recently, ACL primary repair has received increasing attention in ACL treatment. This study aimed to explore the histological characteristics of ACL healing in primary repair and compare its therapeutic and prognostic results with the reconstruction of acute proximal ACL tears. Histological experiments using rabbits and a prospective clinical trial were conducted. We established a rabbit model of ACL primary repair, and histological changes were observed using haematoxylin and eosin (HE) and toluidine blue staining. We performed immunohistochemical analysis of CD34 and S-100 and measured the expression of collagen I and II using qRT-PCR, Western blotting, and immunohistochemistry. The prospective clinical trial involved performing ACL primary repair and reconstruction in patients with acute proximal ACL tears to detect proprioception and evaluate the function of joints. We discovered that primary repair promoted cell proliferation in the tendon-bone transition and ligament portions, reduced osteoarthritis-like pathological changes, and maintained blood vessels and proprioceptors within the ACL. In the clinical trial, primary repair achieved similar therapeutic outcomes, including recovery of knee function and proprioception, in the follow-up period as ACL reconstruction. However, the primary repair had a significantly shorter operative time and lower cost than reconstruction. Therefore, doctors should consider the benefit of primary repair in treating acute proximal ACL tears.

## 1 Introduction

The anterior cruciate ligament (ACL) is most vulnerable to sports injuries (Yang et al., 2019). Compared to the limited effect of conservative treatment in the healing of torn ACLs, the surgical intervention aims to restore knee stability and reduce degeneration changes found in ACL-deficient knees. The management of ACL remnant tissue is essential for clinical outcomes and prognosis. The blood vessels covered by the synovium of the ACL provide a microenvironment for synovial cell proliferation (Takahashi et al., 2016). Furthermore, the proprioceptors in the remnant tissue participate in adjusting muscle movements around the knee joint, thereby accelerating the rehabilitation of knee function (Lee et al., 2015). However, improper handling of the remnant tissue could result in excessive graft volume compared with the space of the intercondylar fossa, resulting in an increased incidence of problematic postoperative loss of extension (Tanabe et al., 2016). Thus, the preservation technique of the remnant tissue deserves proper application in ACL surgery.

Primary repair and reconstruction are representative therapeutic strategies (Achtnich et al., 2016). ACL reconstruction is widely performed and has shown excellent outcomes. However, not all patients experience satisfactory recovery after ACL reconstruction (Kazusa et al., 2013). Therefore, many surgeons have switched their attention to ACL primary repair because of its less invasive nature and potential for self-healing (van der List and DiFelice, 2017; Ahmad et al., 2019; Schneider et al., 2020). Several inherent advantages exist in ACL primary repair, including fewer graft-related complications and maintenance of the original anatomical structures and proprioceptors (Kiapour et al., 2017). Furthermore, recent studies have reported that ACL primary repair is a safe procedure with acceptable overall failure rates among adult patients (Heusdens et al., 2019; Douoguih et al., 2020). However, the clinical efficacy and underlying mechanisms of ACL healing with these two strategies remain controversial.

Although clinical research on ACL primary repair with remnant tissue preservation cannot be neglected, histological research can provide a solid theoretical foundation for further studies. Tendon-bone healing in the transition area and tendon-tendon healing in the ligament portion are essential processes for ACL healing (Maniar et al., 2015). Recent studies have reported that growth factors or stem cells can potentially be administered to accelerate the healing response (Dallo et al., 2017; Xu et al., 2021). Microfracture in ACL primary repair produces a persistent microenvironment for cell proliferation (Achtnich et al., 2016). Both the clinical efficacy and histological characteristics of ACL primary repair should be investigated. However, existing studies have not combined these two aspects.

We hypothesised that primary repair would have similar clinical efficacy as ACL reconstruction, and histological results would provide a foundation for future mechanistic studies. The purpose of this study was to analyse the outcomes of primary repair with remnant tissue preservation in treating acute proximal ACL tears by combining the evaluation of the histological characteristics and clinical efficacy. This study used rabbits to fill the research gaps in ACL primary repair, considering the limitations of conducting histological research using humans. Clinical efficacy was investigated in a prospective clinical trial in patients diagnosed with acute proximal ACL tears with intact remnants, and ACL reconstruction was used as the gold standard.

## 2 Materials and Methods

### 2.1 Histological Experiments

#### 2.1.1 Experimental Animals

Male New Zealand white rabbits aged 6 months and weighing 3.0–3.5 kg were purchased from the Maohua Company (Shenyang, China). Four rabbits were kept in one cage and lived in a controlled environment (22 ± 2°C, 50 ± 10% humidity, and 12 h/12 h light-dark cycle with the light period from 6 a.m. to 6 p.m.). All the rabbits had access to adequate drinking water and were fed ad libitum for 2 weeks to acclimatise them to laboratory conditions. The study followed the 3R rules for animal testing and was approved by the Ethics Committee of Shengjing Hospital.

#### 2.1.2 ACL Primary Repair on Rabbits

There were three groups in the animal experiments: the control group (CG), ACL cutting-off (ACLC) group, and ACL primary repair (ACLP) group. Every 12 rabbits were attributed to each group. Each rabbit was anaesthetised *via* inhalation of a 3% isoflurane-air mixture, and the respiratory rate, heart rate, and body temperature were monitored. The rabbits were then fixed in the supine position, and the skin of both lower limbs was disinfected with iodophor. The articular structures of rabbit knees were exposed by separating the skin and subcutaneous tissues layer-by-layer. The ACL was cut off from the femoral insertion site in the ACLC group. A suture was passed through the ACL bundle several times, and a femoral tunnel was drilled from the femoral footprint, with the knee flexed at 90°. The suture pulled the stump of the ACL through the femoral tunnel, and a cortical bone screw was used to fix the suture to the lateral condyle. The tissue fragments and blood clots in joint cavity was irrigated with normal saline. After the surgery, the animals were returned to their original cages without limitation. The procedure on CG was performed only to expose the knee cavity, which was then closed layer-by-layer. The procedure on ACLC was similary to ACLP, but without repairing the ACL (Supplementary Figure S1).

#### 2.1.3 Sample Collection and Histological Evaluation

The rabbits were sacrificed by overdose anaesthesia 12 weeks after the operation. First, any structures outside the knee joint capsule were removed, maintaining the femoral ligament-tibial complex. The samples were fixed in 4% paraformaldehyde solution for 48 h. Next, the samples were soaked in 10% ethylenediaminetetraacetic acid (EDTA) solution for decalcification for the following 1 month. The EDTA solution was refreshed every 2, 3 days. After decalcification, the samples were dehydrated in a series of ethanol solutions and embedded in paraffin for further experiments. The paraffin-embedded tissues were cut into 5-μM thick sections and stained with haematoxylin and eosin (HE) or toluidine blue stain for evaluation. Finally, the modified Mankin scoring system was used to analyse osteoarthritis (OA)-like changes in the knee joints of the rabbits (Lin et al., 2021).

#### 2.1.4 Immunohistochemistry

After deparaffinisation, rehydration, and washing, enzymatic antigen retrieval of each tissue section was performed at 37°C for 30 min. The sections were treated with 3% H_2_O_2_ in methanol for 10 min to quench endogenous peroxidase activity, and goat serum (5%) was used to block non-specific binding sites. Next, the sections were incubated overnight at 4°C with the following primary antibodies: mouse monoclonal anti-CD34 (1:2,000; 60180-1-Ig, Proteintech, Shenyang, China), mouse monoclonal anti-S100 (1:100; BM0120, BOSTER, Shanghai, China), mouse monoclonal anti-collagen I (1:100; 66761-1-Ig, Proteintech), and mouse monoclonal anti-collagen II (1:300; ab185430, Abcam, Shanghai, China). After washing with phosphate-buffered saline (PBS) on the following day, the sections were incubated with the appropriate biotin-conjugated secondary antibodies at 22 ± 2°C for 30 min. The sections were then incubated with horseradish peroxidase-conjugated streptavidin at 22 ± 2°C for 1 h. Finally, the sections were stained with diaminobenzidine (DAB) and counterstained with haematoxylin. Image Pro Plus version 6.0 software (Media Cybernetics, Rockville, MD, United States) was used to calculate the mean optical density.

#### 2.1.5 Quantitative Reverse Transcription-PCR

Briefly, total RNA was collected from the tendon-bone transition area and articular cartilage using TRIzol^®^ reagent (Thermo Fisher Scientific, Waltham, MA, United States). A PrimeScript RT Reagent Kit with gDNA Eraser (Takara, Shiga, Japan) was used to synthesise complementary DNA, and Quantitative Reverse Transcription-PCR (qRT-PCR) was performed using an ABI Prism 7500 Fast Real-Time PCR System (Thermo Fisher Scientific) and SYBR Premix Ex Taq II (Takara). The 2^−ΔΔCT^ method was used to evaluate the relative expression of *GAPDH* as the reference. Primers were designed and synthesised by Sangon Biotech (Shanghai, China) (Table 1).

**TABLE 1:** Primers used in the study.

Primers	5′ to 3′	3′ to 5′
Collagen I	GCC​ATC​AAG​GTC​TTC​TGC​G	GAA​CTG​GAA​GCC​ATC​GGT​C
Collagen II	ACA​CTG​CCA​ACG​TCC​AGA​TG	GTG​ATG​TTC​TGG​GAG​CCC​TC
*GAPDH*	GGG​AAG​CTG​GTC​ATC​AAC​GG	GTA​CTC​GGC​ACC​AGC​ATC​AC

#### 2.1.6 Western Blotting

Briefly, proteins were collected from the tendon-bone transition area and articular cartilage using radioimmunoprecipitation assay (RIPA) lysis buffer (P0013C, Beyotime, Shenyang, China) supplemented with 1% phenylmethylsulfonyl fluoride (PMSF; ST506, Beyotime). A BCA protein assay kit (P0010; Beyotime) was used to quantify the proteins, and 30 μg protein per sample was subjected to polyacrylamide gel electrophoresis. Next, the proteins were transferred onto polyvinylidene difluoride (PVDF) membranes using wet blotting. The membranes were incubated overnight at 4°C with the following primary antibodies after blocking non-specific binding sites: mouse monoclonal anti-collagen I (1:3,000; 66761-1-Ig, Proteintech), mouse monoclonal anti-collagen II (1:3,000; ab185430, Abcam, United States), and mouse monoclonal GAPDH (1:1,000; 60004-1-Ig; Proteintech). The membranes were then incubated with horseradish peroxidase-conjugated secondary antibodies at 22 ± 2°C for 2 h. Enhanced chemiluminescence reagent was used to detect immunoreactivity. Finally, Image Pro Plus version 6.0 software (Media Cybernetics) was used to calculate the band intensities.

### 2.2 Clinical Trial

#### 2.2.1 Patient Information and Inclusion and Exclusion Criteria

Our study followed the latest Strengthening the Reporting of Cohort Studies in Surgery guidelines (Agha et al., 2019). Furthermore, the study protocol was approved by the ethics committee and prospectively registered in the Chinese Clinical Trial Registry (https://www.chictr.org.cn/showproj.aspx?proj=124186, ChiCTR2100045145). Informed consent was obtained from all patients. Baseline information, including clinical history, surgical date, nursing, and imaging data, was collected from the hospital information system.

**FIGURE 1:**
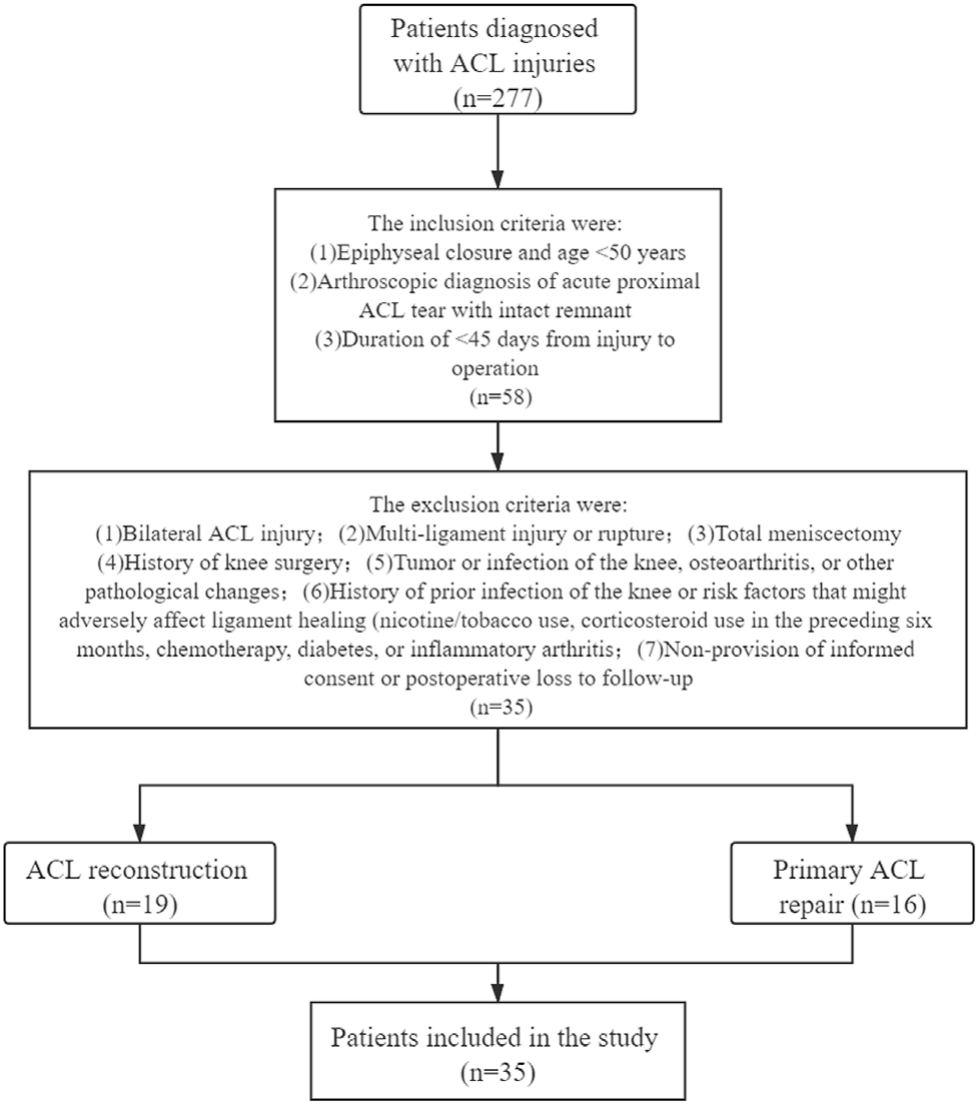
Flowchart of inclusion and exclusion criteria for patients in the ACL reconstruction and primary repair groups.

The inclusion criteria were epiphyseal closure, age <50 years, arthroscopic diagnosis of acute proximal ACL tear with an high quality remnant, and an interval of 45 days between the injury and surgery. The exclusion criteria were: 1) bilateral ACL injury; 2) multi-ligament injury or rupture; 3) total meniscectomy; 4) history of knee surgery; 5) tumour or infection of the knee, OA, or other pathological changes; 6) history of prior infection of the knee or risk factors that might adversely affect ligament healing (nicotine/tobacco use, corticosteroid therapy in the preceding 6 months, chemotherapy, diabetes, or inflammatory arthritis); and 7) non-provision of informed consent or postoperative loss to follow-up. The inclusion and exclusion criteria and flow chart of the clinical trial are shown in Figure 1.

To fully respect the patient’s right to informed consent, we explained the advantages and disadvantages of ACL primary repair and ACL reconstruction to each patient and obtained their authorisation and consent preoperatively. Each patient decided on the surgical plan and signed a surgical consent form before the operation.

#### 2.2.2 Surgical Technique

All ACL primary repairs (Group P) and ACL reconstructions (Group R) were performed by the same senior surgeon. After successful induction of anaesthesia, the patient was placed in the supine position on the operation table. The affected knee was prepared and draped for arthroscopy. Equipment and implants in the standard knee arthroscopy set were used. A scalpel was used to make a 0.4 cm longitudinal incision, and anterolateral and anteromedial portals were created. A probe was used to explore the joint cavities sequentially. Finally, the proximal ACL tear was diagnosed. The ACL remnant tissue quality was evaluated by sherman scoring system before surgery and Takeshi scoring system during ACL surgery (Sherman et al., 1991; Muneta et al., 2016).

##### 2.2.2.1 ACL Primary Repair Procedure

The ACL primary repair procedure is shown in the Supplementary Video S1. Briefly, the degree of ACL damage was assessed *via* arthroscopy. Next, an AR-7200 suture (Smith & Nephew, London, UK) was passed through the ACL bundle *via* a Scorpion Suture Passer (Smith & Nephew). The ACL bundle was retracted to expose the ACL footprint on the lateral femoral condyle. A planer was used to trim the damaged joint surfaces, and a microfracture device (Smith & Nephew) was used to drill a hole to freshen the femoral footprint area and promote the healing of the ACL bundle.

A femoral tunnel was drilled from the femoral footprint, with the knee flexed at 90°. Next, the AR-7200 sutures were retrieved through the medial portal and passed through the core hole of the PushLock anchor (Smith & Nephew). The PushLock anchor was deployed into the femoral tunnel, with the knee flexed at 90°.

The free end of the AR-7200 suture was cut using an open-ended suture cutter (Smith & Nephew), and a probe was used to assess tension in the ACL bundle. ACL impingement should not be observed on arthroscopy. Next, an intraoperative Lachman test was performed to confirm minimal anteroposterior translation after primary repair. Intra-articular electrocautery was used to achieve haemostasis. The tissue fragments and blood clots in joint cavity was irrigated with normal saline. Finally, the arthroscopic equipment was removed, and the surgical portal was closed layer-by-layer (Supplementary Figure S2).

##### 2.2.2.2 ACL Reconstruction Procedure

The semimembranosus and semitendinosus were harvested from patients. These ligaments were folded, quadrupled, and stitched using an AR-7200 suture (Smith & Nephew) following a Bunnell-type pattern and immersed in gentamicin saline for further use. After installing the tibial guide frame, the arthroscope was used to determine the inner port of the tibial tunnel 7 mm from the anterior edge of the posterior cruciate ligament and 3 mm behind the midpoint of the ACL implantation point. The outer port of the tibial tunnel was located 1.5 cm posterior to the tibial tubercle and 1 cm above the pes anserinus. The tibial tunnel was created based on the diameter of the graft ligaments. The anteromedial auxiliary portal was established to drill the femoral tunnel at the femoral footprint of the ACL. The femoral tunnel was created 1 mm from the posterior femoral cortex and was 3 cm deep.

Another suture was placed at the proximal end of the remnant with a suture hook (Smith & Nephew). The free ends of the sutures were retrieved through the anteromedial portals. The remnant tissue of the ACL bundle was preserved and protected using sutures during ACL reconstruction. A pulling suture was used to insert the graft into the tibial and femoral tunnels. An EndoButton loop steel plate (Smith & Nephew) was used to fix and tighten the looped graft ligament. A squeezing screw was used to fix the tibial end of the graft with the knee at a 20° extension.

The proximal ACL remnant and the articular portion of the graft were sutured using an AR-7200 suture when the tibia was completely fixed. The free end of the AR-7200 suture on the ACL remnant bundle was passed through the core hole of the PushLock anchor (Smith & Nephew). The PushLock anchor was deployed into the femoral tunnel with the knee at 90° flexion. The free end of the AR-7200 suture was cut using an open-ended suture cutter (Smith & Nephew).

Mobility of the affected knee was tested from 0° to 130°. Graft impingement should not be observed under arthroscopy, and the change in graft length should be <2 mm. A probe was used to assess tension in the ACL bundle, and an intraoperative Lachman test was performed to confirm the minimal anteroposterior translation. Intra-articular electrocautery was used to achieve haemostasis. The tissue fragments and blood clots in joint cavity was irrigated with normal saline. Finally, the arthroscopic equipment was removed, and the surgical portal was closed layer-by-layer (Supplementary Figure S3).

#### 2.2.3 Postoperative Management

Postoperatively, all patients underwent magnetic resonance imaging and were guided to undergo rehabilitation exercises (Supplementary Figure S4). The patients wore limb braces, and drainage tubes were removed after approximately 24 h. The brace was fixed at 0–30° within 1 week after the operation. After 1 month, the range of motion of the knee joint was expected to reach 90–110° and allow for a gradual transition to total weight-bearing.

#### 2.2.4 Clinical Efficacy Assessment

The Lysholm score and International Knee Documentation Committee (IKDC) score were used to evaluate the basic function of the knee joint subjectively (Chai at al., 2019; Mouarbes et al., 2019), and the knee joint passive relaxation test (Kneelax) was used to evaluate the stability of the knee joint objectively. The threshold to detect passive motion (TDPM) test, joint position reproduction (JPR) test, and foot pressure analysis were used to evaluate proprioception.

##### 2.2.4.1 Knee-Joint Assessment Using Kneelax 3

Kneelax 3 (Shanghai Huanxi Medical Instrument Co., Ltd., Shanghai, China) was used to evaluate the stability of the knee joint. The distance of the anterior tibial displacement per millimetre under 132 N tension was recorded for statistical analysis.

##### 2.2.4.2 TDPM

The TDPM was evaluated using a continuous passive motion (CPM) machine. The patient was placed in a recumbent position with earmuffs and eyeshades to isolate the visual and auditory sensations. The lower limbs were placed in the CPM machine. The machine started to drive at 15° and an angular speed of 0.5°/s. When the patient perceived lower-limb movement, the machine was stopped. Simultaneously, the patient indicated the direction of the movement. This test was repeated three times, and the average time was recorded as the TDPM value.

##### 2.2.4.3 JPR

JPR tests were performed under the same conditions as mentioned in Section 2.2.4.2. The limbs were placed at various measurement angles (30°, 60°, and 90°) and rested for 10 s. The CPM machine was stopped when the patient perceived the measurement angle. The test was repeated three times to obtain the average difference between the perceived and measurement angles. Finally, the JPR value was obtained by calculating the average difference for each angle.

##### 2.2.4.4 Foot Pressure Analysis of Gait

A foot pressure analysis system was used to collect data on the walking parameters of patients. It analysed the proportion of each stage of the walking support phase and evaluated the stability of the centre of weight distribution of patients. The gait system divides the single-foot support phase into four phases: the initial contact phase (ICP), forefoot contact phase (FFCP), foot flat phase (FFP), and forefoot push-off phase (FFPOP) (Supplementary Figure S5).

### 2.3 Statistical Analysis

The Shapiro–Wilk test was used to evaluate the normality, and the Levene test was used to evaluate the homogeneity of variance before assessing the differences between groups. The Student’s *t*-test was used to analyse normally distributed data with homogeneity of variance, and the results were expressed as the mean ± standard deviation. Otherwise, the Wilcoxon rank-sum test was used, and the results were expressed as medians with interquartile ranges. Categorical data were analysed using the chi-square test. Statistical analyses were performed using SPSS version 21.0 (IBM Corp., Armonk, NY, United States). *p* < 0.05 was considered statistically significant.

## 3 Results

### 3.1 Primary Repair Promotes Cell Proliferation in Tendon-Bone Transition and Ligament Portions

**FIGURE 2:**
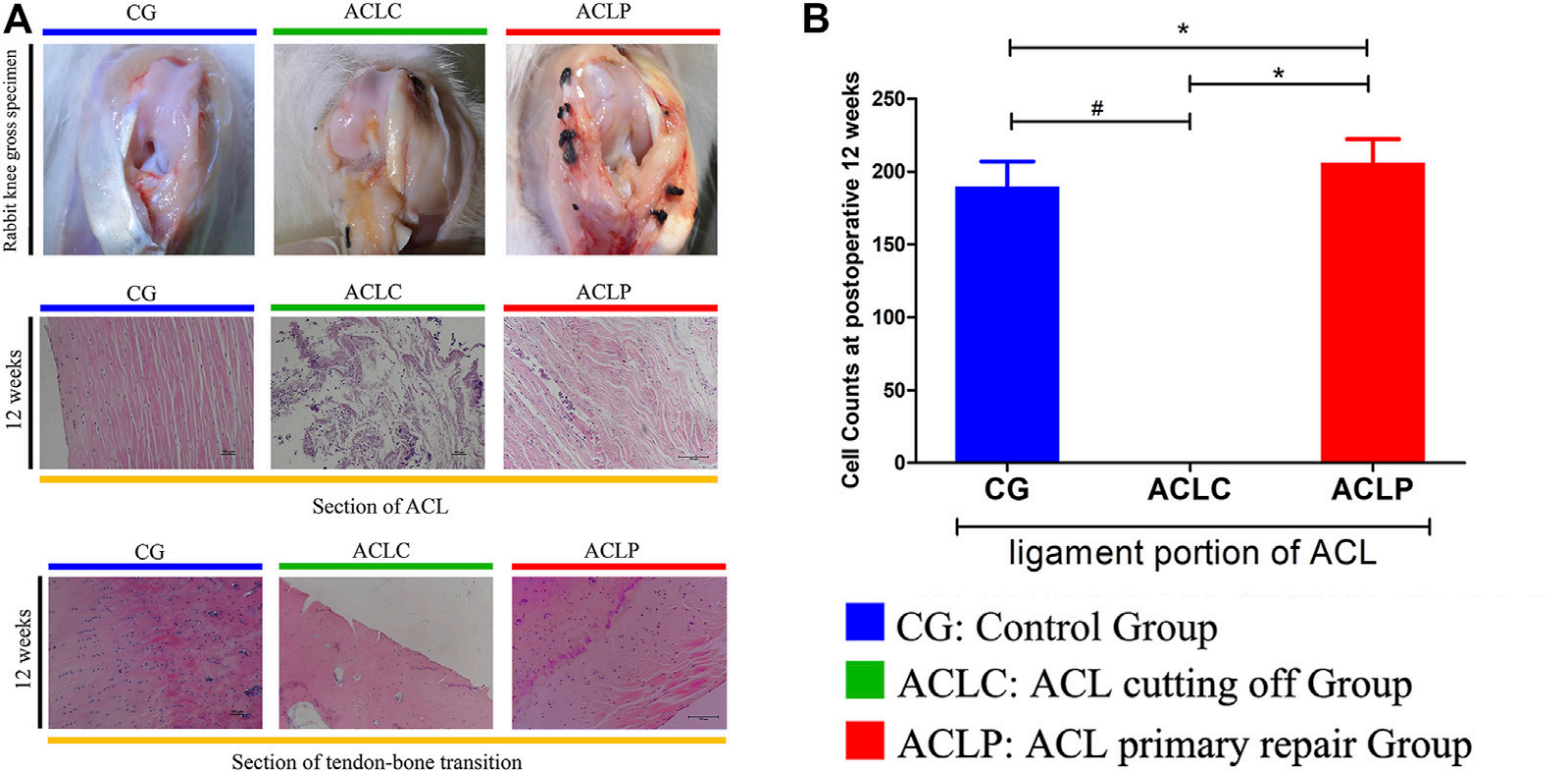
**(A)** The gross specimen of the rabbit knee and HE staining of the ACL portion and tendon-bone transition area at postoperative week 12. **(B)** Cell Counts of ACL portion. ^#^: *p* < 0.05 versus ACLC group; *: *p* < 0.05 versus ACLP group. *n* = 12 in each group. Data are expressed as mean ± SD.

As shown in Figure 2A, the normal ACL has a smooth surface and is covered by synovial tissue with tiny blood vessels. The ligament tissue of the ACLC group was almost completely absorbed at postoperative week 12. Synovial-like tissue could be seen on the ligament surface in the ACLP group without apparent scar formation at the healing site. At postoperative week 12, the tendon-bone junction was tighter, the tide line was wide, and more tissue interpenetration could be seen in the ACLP group. Increased nucleus density was observed in the ligament portion of the ACLP group at postoperative week 12. The cell counts of the ACLP group were significantly increased at postoperative week 12 (*p* < 0.05), whereas 0 cell count was observed in the ACLC group (Figure 2B). Additionally, no healing response was observed in the tendon-bone transition area of the ACLC group.

### 3.2 Primary Repair Reduces OA-like Pathological Changes

**FIGURE 3:**
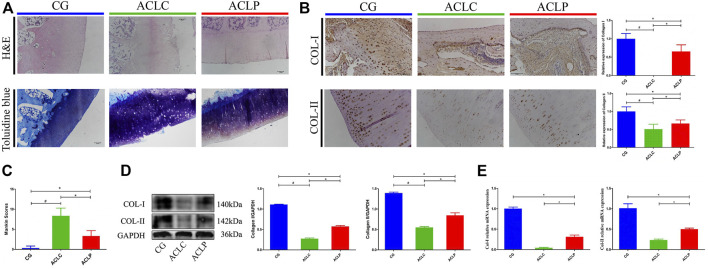
**(A)** HE and toluidine blue staining of articular cartilage in the rabbit knee. **(B)** Immunohistochemistry of collagen I and II in articular cartilage. **(C)** Mankin scores of each group. **(D)** Western blotting of collagen I and II in articular cartilage. **(E)** Relative mRNA expression of collagen I and II in articular cartilage. ^#^: *p* < 0.05 versus ACLC group; *: *p* < 0.05 versus ACLP group. *n* = 12 in each group. Data are expressed as mean ± SD.

OA-like changes are long-term pathological features worthy of attention after ACL healing. Figure 3A showed the H&E and toluidine blue staining of the rabbit knee surface, respectively. After cutting the ligament in the ACLC group, OA-like changes such as local cartilage damage, empty cartilage lacunae, and light staining of the extracellular matrix could be observed. However, the ACLP group had a relatively smooth cartilage surface, a higher density of chondrocytes. ACLP had an significant decreased Mankin scores compared to ACLC (Figure 3C, *p* < 0.05). The relative expressions of collagen I in the transition area and collagen II in articular cartilage were evaluated using immunohistochemistry (Figure 3B), Western blotting (Figure 3D) and qPCR (Figure 3E). Increased expression of collagen I and II was observed in the ACLP group (*p* < 0.05). This may suggest that the stability restored by primary repair reduced the process of knee joint degeneration.

### 3.3 Primary Repair Protects Blood Vessels and Proprioceptors Within the ACL

**FIGURE 4:**
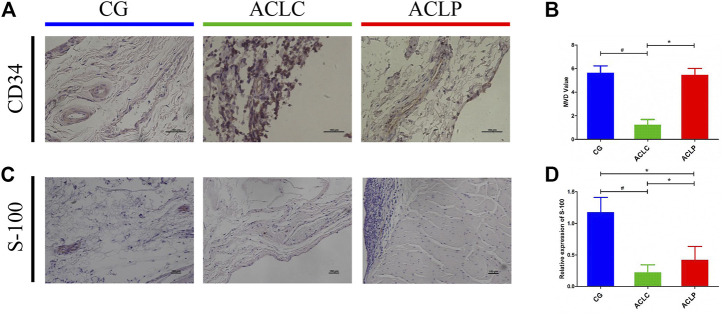
**(A)** Immunohistochemistry of CD34. **(B)** MVD value of each group according to immunohistochemistry results of CD34. **(C)** Immunohistochemistry of S-100 protein. **(D)** Statistical analysis of S-100 protein. ^#^: *p* < 0.05 versus ACLC group; *: *p* < 0.05 versus ACLP group. *n* = 12 in each group. Data are expressed as mean ± SD.

CD34 is a protein characteristic of the vascular structure. The expression of CD34 in the ACLP group was significantly higher than that in the ACLC group. The statistical value of microvessel density (MVD) was used as the evaluation standard for ligament blood supply (Nayak et al., 2020). The MVD of the ACLP group was significantly higher than that of the ACLC group (Figures 4A,B, *p* < 0.05). S-100 protein is a marker of proprioceptors in the ligaments. The expression of S-100 in the ACLP group was significantly higher than that in the ACLC group (Figures 4C,D, *p* < 0.05).

### 3.4 No Difference Exists in Preoperative Baseline Information

**FIGURE 5:**
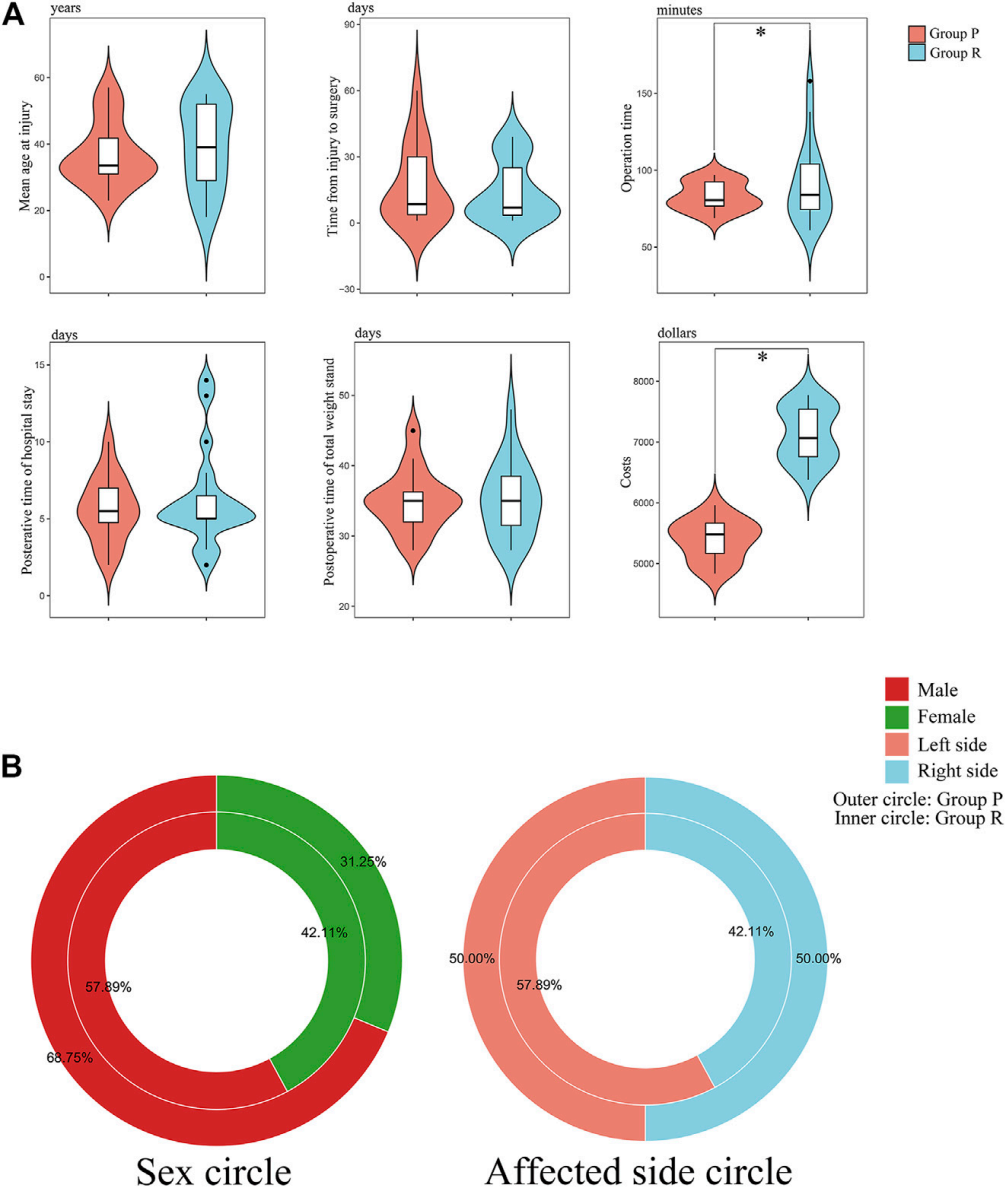
Visual chart of baseline characteristics and surgical data of patients. **(A)** Violin plots of key data: age at injury; time from injury to surgery; operation time; postoperative time of hospital stay; postoperative time of total weight stand; and costs. *: *p* < 0.05 versus Group P. **(B)** Donut diagram of sex and affected side distribution.

Group P contained 16 patients, and Group R had 19 patients. No significant differences were observed in the preoperative distributions of sex, age, affected knee, ASA grade, and comorbidities between the two patient groups (Table 2). It means that the these groups were at the same baseline before surgery and postoperative outcomes were comparable. We also created a visual representation of the data from Table 2 (Figure 5).

**TABLE 2:** Baseline characteristics of patients.

Baseline characteristics		Group P (*n* = 16)	Group R (*n* = 19)	*p* value
Gender	Male	11 (68.75%)	11 (57.89%)	0.508
	Female	5 (31.25%)	8 (42.11%)	
Mean age at injury (years)		37.00 ± 9.66	39.53 ± 12.81	0.521
Laterality of injury	Left	8 (50.00%)	11 (57.89%)	0.640
	Right	8 (50.00%)	8 (42.11%)	
ASA grade	I	11	13	0.983
	II	5	6	
	III	0	0	
	IV	0	0	
Comorbidities	Cardiovascular system disease	3	4	0.865
	Respiratory system disease	1	1	0.900
	Urological system disease	1	0	0.269
	Thrombogenesis	1	1	0.900
	Metabolic disorder	1	1	0.900

ASA, american society of anaesthesiologists.

### 3.5 ACL Primary Repair Substantially Decreased the Operation Time and Costs

Surgery-related data are presented in Table 3. There were no significant differences in the time from injury to operation, anaesthesia method, time to partial or total standing, postoperative length of stay, and complications. However, Group P had less operative time and costs than Group R (*p* < 0.05).

**TABLE 3:** Surgical data.

Parameters		Group P (*n* = 16)	Group R (*n* = 19)	*p* value
Time from injury to operation (days)		16.5 (9–35.75)	15 (9–35)	0.829
Method of anaesthesia	General anaesthesia	6	6	0.713
	CSEA	10	13	
Operation time (min)		73.8 (68.9–78.6)	85.2 (78.4–91.9)	0.007
Median time to partial standing (days)		2 (2–3)	2 (2–3)	>0.999
Median time to total standing (days)		34.94 ± 4.31	35.11 ± 5.28	0.920
Postoperative length of stay (days)		5.63 ± 2.09	6.26 ± 3.11	0.490
Postoperative complications		0	0	
Cost (thousand dollars)		5.39 ± 0.33	7.14 ± 0.45	<0.001

### 3.6 Group P Patients Have Similar Knee Function as Group R

**FIGURE 6:**
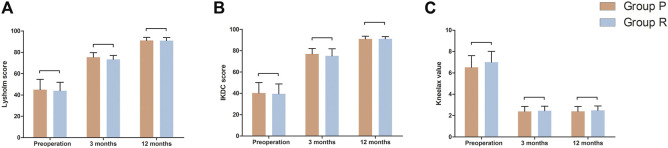
Function measurements of affected knee joint in Group P and Group R. **(A)** Lysholm score. **(B)** IKDC score. **(C)** Kneelax value. Data are expressed as mean ± SD.

The follow-up period for the 35 patients was 12 months. The preoperative and postoperative Lysholm and IKDC scores of the knee joints in the two groups are shown in Table 4. The postoperative Lysholm and IKDC scores increased significantly compared with the corresponding baseline preoperative scores in both groups (*p* < 0.05). There were no significant differences in the function of the knee joint preoperatively and at 3 and 12 months postoperatively between both groups (Table 4). The preoperative and postoperative distances of anterior tibial displacement assessed using Kneelax in both groups are shown in Table 4. There were no significant differences in the anterior tibial displacement between the groups at 3 and 12 months postoperatively, and adequate anterior and posterior stability was achieved (Table 4). Figure 6 is a visual display of the data in Table 4.

**TABLE 4:** Follow-up and function measurements.

Parameters		Group P (*n* = 16)	Group R (*n* = 19)	*p* value
Age at the latest follow-up (years)		34.5 (32–44.25)	40 (30–54)	0.446
Lysholm score	Preoperatively	44.87 ± 9.66	43.89 ± 8.05	0.745
	3 months postoperatively	75.38 ± 4.33	73.31 ± 3.71	0.139
	12 months postoperatively	91.06 ± 2.91	90.89 ± 2.94	0.867
IKDC score	Preoperatively	40.25 ± 9.90	39.47 ± 9.38	0.813
	3 months postoperatively	76.88 ± 5.24	75.05 ± 6.73	0.385
	12 months postoperatively	90.95 ± 2.62	90.99 ± 2.21	0.963
Kneelax value (mm)	Preoperatively	6.52 ± 1.09	7.00 ± 1.01	0.185
	3 months postoperatively	2.38 ± 0.49	2.45 ± 0.43	0.671
	12 months postoperatively	2.39 ± 0.47	2.48 ± 0.42	0.548

### 3.7 Group P Obtained Similar Proprioception Scores as Group R

**FIGURE 7:**
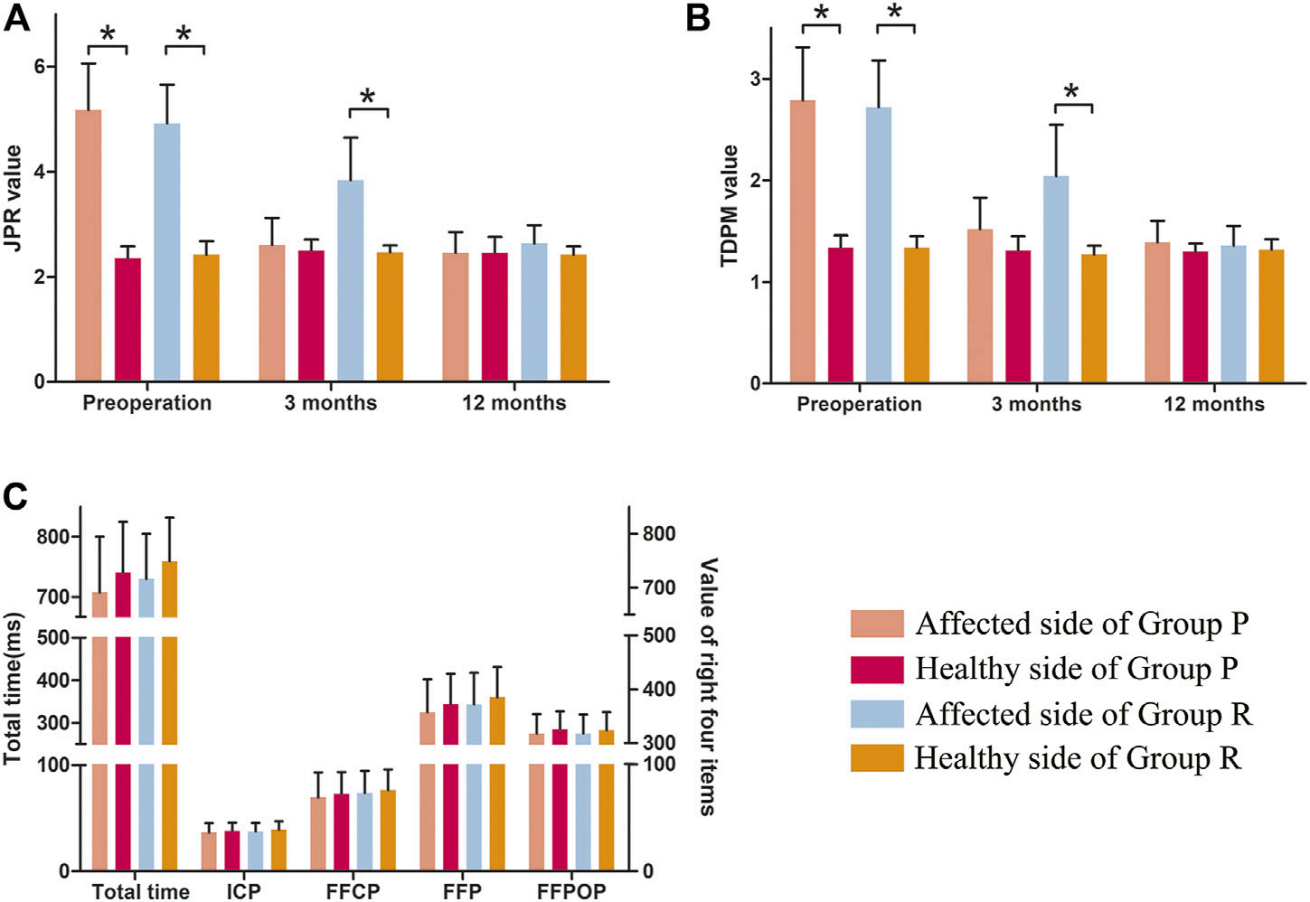
Proprioception of the knee in Group P and Group R. **(A)** JPR value. **(B)** TDPM value. **(C)** Total time, ICP, FFCP, FFP, and FFPOP. *: *p* < 0.05 versus healthy side in each group. Data are expressed as mean ± SD.

A comparison of proprioceptive assessments between the two groups is shown in Table 5 and Figure 7. Table 5 is for comparison between groups (Group P verus Group R). While Figure 7 is a visual display of the same data for intra-group comparison (Affected side versus Healthy side). Group P obtained similar proprioception function as Group R at 3 and 12 months postoperatively, which was reflected by TDPM, JPR, ICP, FFCP, FFP, and FFPOP (Table 5). What stands out in the Figure 7 is an significant increased JPR and TDPM preoperative value in Affected side of each group, compared to the Healthy side (JPR: Figure 7A; TDPM: Figure 7B, *p < 0.05). However, no difference in JPR and TDPM value could be obsevered between Affected and Healthy side at postoperative 12 months. Further, Figure 7C illustrated no statistical difference existed in total time, ICP, FFCP, FFP, and FFPOP (Affected side versus Healthy side).

**TABLE 5:** Distribution of proprioceptive parameters of the knee.

Parameters		Group P (*n* = 16)	Group R (*n* = 19)	*p* value
TDPM value of affected side	Preoperatively	2.79 ± 0.52	2.72 ± 0.46	0.701
	3 months postoperatively	2.01 ± 0.16	2.04 ± 0.51	0.769
	12 months postoperatively	1.39 ± 0.21	1.36 ± 0.19	0.652
TDPM value of healthy side	Preoperatively	1.34 ± 0.12	1.34 ± 0.11	0.920
	3 months postoperatively	1.31 ± 0.14	1.27 ± 0.09	0.330
	12 months postoperatively	1.30 ± 0.08	1.32 ± 0.10	0.488
JPR value of affected side	Preoperatively	5.18 ± 0.88	4.92 ± 0.74	0.359
	3 months postoperatively	3.67 ± 0.55	3.84 ± 0.81	0.467
	12 months postoperatively	2.46 ± 0.39	2.64 ± 0.34	0.155
JPR value of healthy side	Preoperatively	2.46 ± 0.22	2.42 ± 0.26	0.468
	3 months postoperatively	2.50 ± 0.21	2.47 ± 0.13	0.646
	12 months postoperatively	2.46 ± 0.30	2.42 ± 0.16	0.606
Total time (ms)	Affected side	707.56 ± 92.61	729.89 ± 75.48	0.437
	Healthy side	741.06 ± 84.18	759.42 ± 72.59	0.493
ICP	Affected side	36.56 ± 8.61	37.53 ± 7.95	0.733
	Healthy side	38.19 ± 7.39	39.32 ± 7.89	0.667
FFCP	Affected side	69.75 ± 23.32	73.84 ± 20.70	0.586
	Healthy side	72.88 ± 20.46	76.53 ± 19.44	0.592
FFP	Affected side	325.69 ± 76.85	343.42 ± 74.00	0.493
	Healthy side	344.75 ± 70.32	360.63 ± 70.12	0.510
FFPOP	Affected side	275.56 ± 45.02	275.58 ± 44.04	0.999
	Healthy side	285.25 ± 42.18	282.95 ± 42.53	0.874

## 4 Discussion

ACL primary repair is a surgical technique used to treat acute proximal ACL tears and is currently being refined. In this study, we performed a series of basic experiments and a prospective clinical trial on ACL primary repair. The basic experiments revealed the advantage of primary repair in promoting healing in the tendon-bone transition area and ligament portion. The clinical trial suggested that primary repair could obtain a similar postoperative prognosis as ACL reconstruction in treating acute proximal ACL tears.

The ACL healing process starts with an inflammatory response, followed by cell and extracellular matrix proliferation in several weeks (Nyland et al., 2020). The healing response in the tendon-bone transition area and ligament portion directly influences the stability of the repaired ACL (Woodall et al., 2018). Therefore, remnant tissue preservation and microfracture are applied in ACL primary repair to provide a microenvironment for tissue cell proliferation (Ouanezar et al., 2018). Interestingly, our results illustrated that primary repair achieved a good degree of healing in the tendon-bone transition area and ligament portion in an animal model, which was the foundation of the therapeutic effects achieved in the clinical trial.

The microvessels provide sufficient blood supply for the generation of tissue cells, and proprioceptors regulate deep sensations and muscle strength of the knee joint, which are also histological indices of ACL healing evaluation (Takahashi et al., 2016). CD34 is the most sensitive marker of blood vessels in various organs (Jiang et al., 2021). In this study, cutting the ACL resulted in the disintegration and absorption of ACL remnants, thereby reducing the microvessels. However, typical vascular structures, including many red blood cells, were observed in the ACLP group. This shows that the primary repair effectively preserves the vascular structure in the stump and has a significant effect on tissue healing. S-100 is a proprioceptor marker protein in ligaments (Zhang et al., 2016). Immunohistochemical staining confirmed that neural structures were mainly present in the synovial tissue surrounding the ligament. This indicates that proprioceptive receptors in the ACL can be retained after the primary repair. Therefore, we evaluated the proprioceptive function of primary repair compared with that of reconstruction in the clinical trial. Primary repair achieved similar results as reconstruction in proprioceptive function tests, including TDPM, JPG, ICP, FFCP, FFP, and FFPOP. The results for the microvessels and proprioceptors support each other.

ACL ruptures are strongly associated with an increased risk of post-traumatic OA (Jungmann et al., 2016). On the one hand, the OA-like pathological change was detected. On the other hand, knee function tests were performed during the follow-up period of clinical trials, and a contrasting trend of OA-like pathological changes was observed between the ACLC and ACLP groups. The high Mankin scores and the increased collagen I and II levels in qPCR, Western blotting, and immunohistochemistry indicated that primary repair prevented OA characteristic occuring in the animal model. Furthermore, upward trends existed in the Lysholm score, IKDC score, and Kneelax value with increasing follow-up time. Therefore, ACL primary repair could restore knee stability.

Indications are worth considering before ACL primary repair. Our results show three key indications: the acute phase of history (no more than 2 months), proximal tears, and intact remnant tissue. If these indications are not met, they will eventually be reflected in the quality of the remnant, such as excessive absorption or insufficient tension to support primary repair (Haviv et al., 2019). Although primary repair has a narrow range of indications, it can still bring considerable benefits to target patients compared with ACL reconstruction. First, it achieved a similar prognosis in the follow-up period as ACL reconstruction. Second, primary repair has the advantages of a shorter operation time, operation-related trauma, and costs. Third, patients have an opportunity to undergo reconstruction surgery rather than more difficult revision surgery if primary repair failure occurs. These advantages of primary repair were reflected in the clinical trial of our study.

Our study has some limitations. First, the potential molecular mechanism of the healing response in the tendon-bone transition area and ligament portion was not investigated. Second, randomisation was not performed to respect patients’ right to informed consent. Third, the exclusion criteria may have introduced selection bias in our results. Due to financial and technical constraints, we have not been able to achieve ACL reconstruction in an animal model. Future research can improve this defect to obtain more in-depth research conclusion. Furthermore, we will continue to follow up the selected patients and pay attention to the occurrence of long-term complications, such as graft fracture, occurrence of secondary injury, etc.

In conclusion, we investigated the role and prognosis of ACL primary repair in treating acute proximal ACL tears using histological studies and a clinical trial. ACL primary repair promoted the healing response in the tendon-bone transition area and ligament portion, with increased blood vessels and proprioceptors. The histological results served as the foundation of the clinical trial in our study. The trial revealed that ACL primary repair achieved similar levels of therapeutic performance as ACL reconstruction, including recovery of function, stability, and proprioception in the knee joint. Therefore, we believe that ACL primary repair could be a clinical alternative to the current reconstruction technique to treat acute proximal ACL tears.

## Data Availability

The original contributions presented in the study are included in the article/Supplementary Material, further inquiries can be directed to the corresponding author.

## Supplementary Material

Supplementary Figure S1 The surgery procedures of primary repair performed on the rabbit.

Supplementary Figure S2 Operative procedure of ACL primary repair. **(A)** The ruptured ACL must be acute and proximal, and the remnant must not have been absorbed to apply this technique. **(B)** The proximal end of the ACL was treated with a microfracture to provide adequate blood supply for primary repair. **(C)** AR-7200 was used to suture the ACL approximately 2 cm from the broken end. **(D)** A PushLock anchor was used to fix the ACL remnant at the attachment imprint.

Supplementary Figure S3 Operative procedure for ACL reconstruction. **(A)** The ACL rupture must be an acute proximal ACL tear and the remnant must not have been absorbed to apply this technique. **(B)** After installing the tibial guide frame, the arthroscope was used to determine the inner port of the tibial tunnel at 7 mm from the anterior edge of the posterior cruciate ligament and 3 mm behind the midpoint of the ACL implantation point. **(C)** The femoral tunnel was created 1 mm from the posterior femoral cortex and 3 cm deep. **(D)** Another suture was placed at the proximal end of the remnant with a suture hook. **(E)** A PushLock anchor was used to fix the remnant of the ACL at the attachment imprint.

Supplementary Figure S4 Preoperative and postoperative magnetic resonance and arthroscopic images.

Supplementary Figure S5
**(A)** CPM machine. **(B–D)** Schematic diagram of the gait plantar pressure analysis system.

## Abbreviations

FFCP, forefoot contact phase; FFP, foot flat phase; FFPOP, forefoot push-off phase; ICP, initial contact phase; JPR, joint position reproduction; TDPM, threshold to detect passive motion.
