# Supplementary figures and images for: Primary Repair for Treating Acute Proximal Anterior Cruciate Ligament Tears: A Histological Analysis and Prospective Clinical Trial
Source: Front Bioeng Biotechnol. 2022 May 27;10:913900. doi: 10.3389/fbioe.2022.913900 (PMC9195517; doi:10.3389/fbioe.2022.913900)

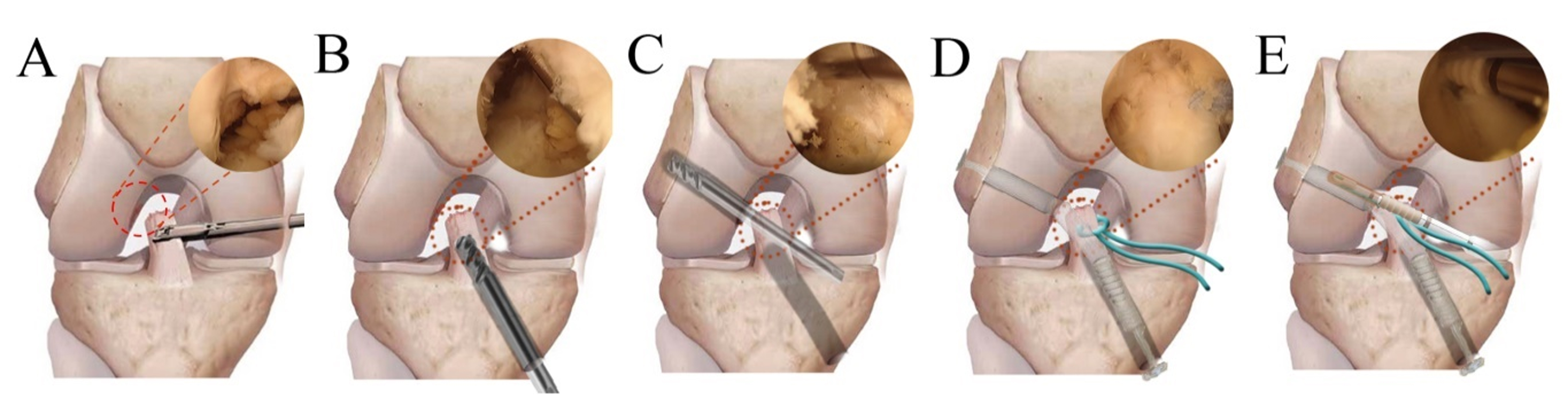

Supplement: Supplementary file 1 [file Image3.JPEG]

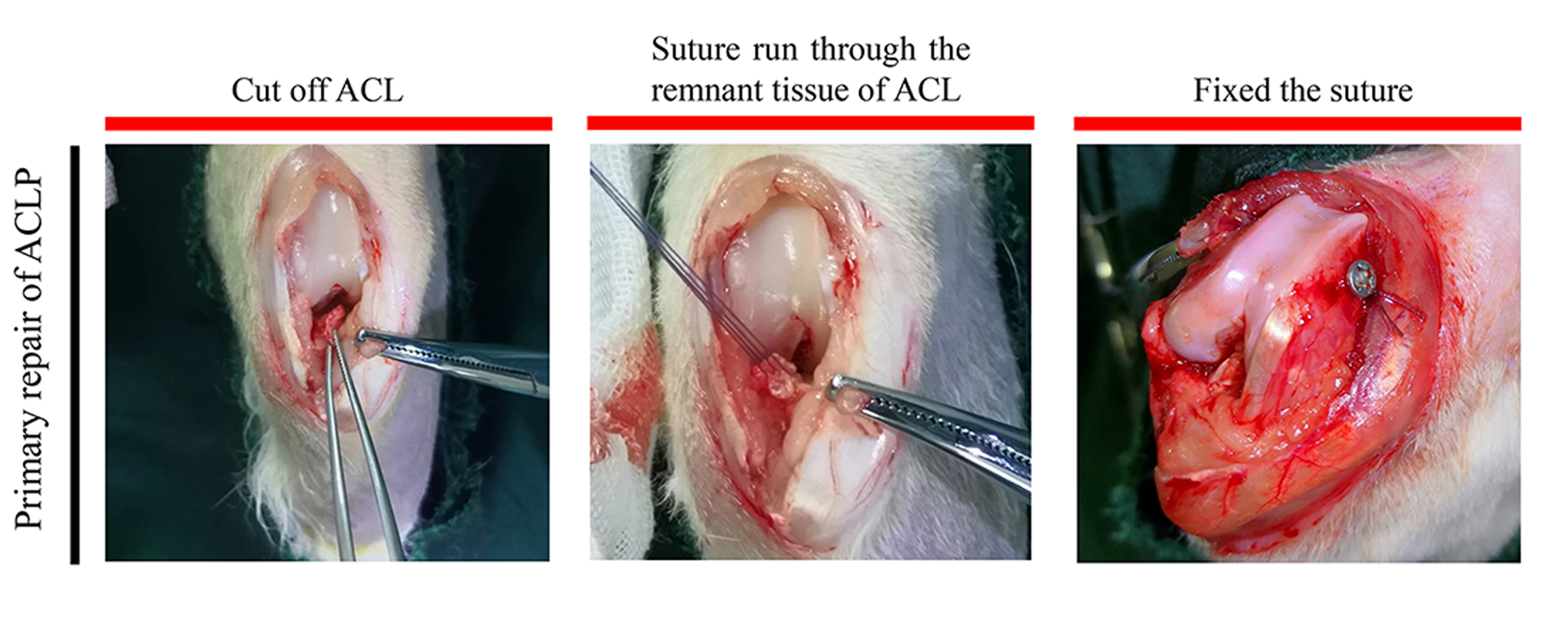

Supplement: Supplementary file 3 [file Image1.JPEG]

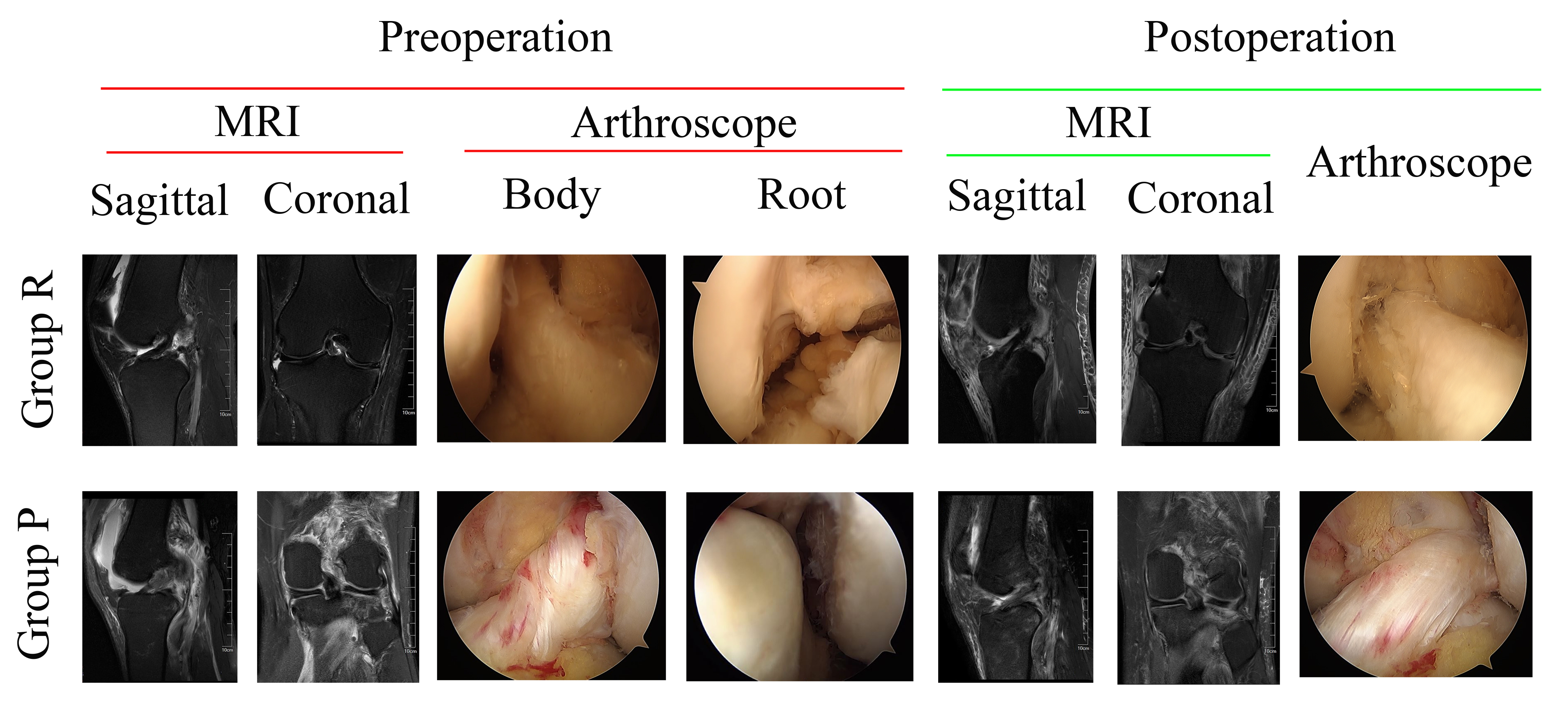

Supplement: Supplementary file 4 [file Image4.JPEG]

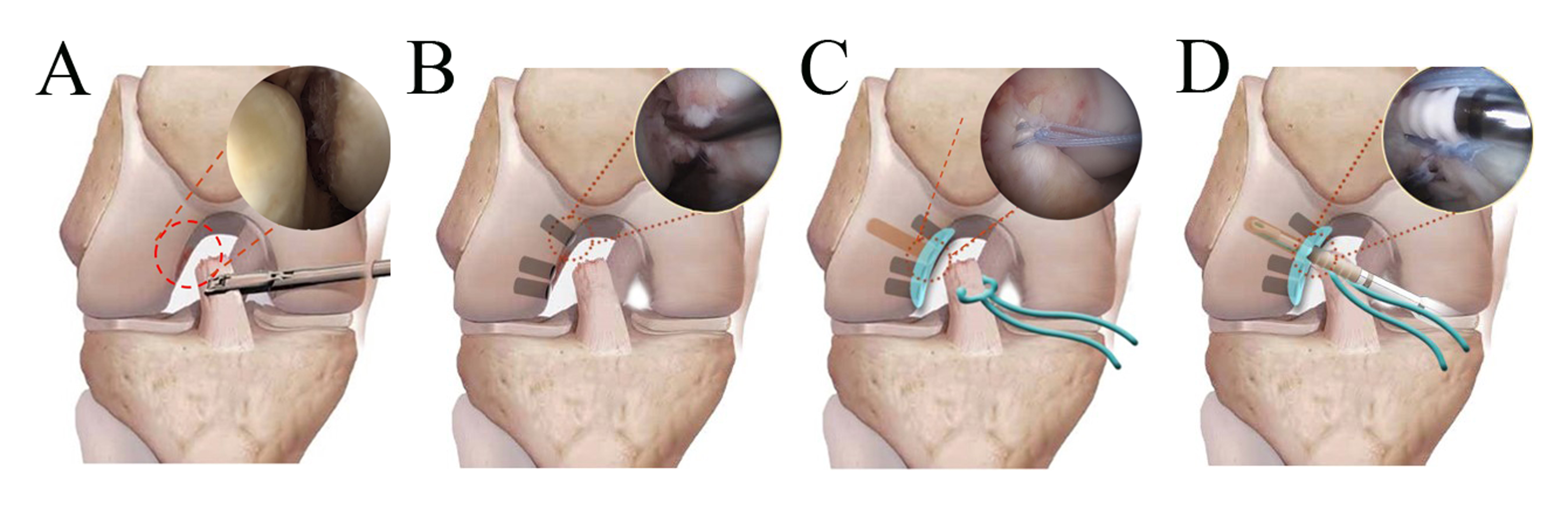

Supplement: Supplementary file 5 [file Image2.JPEG]

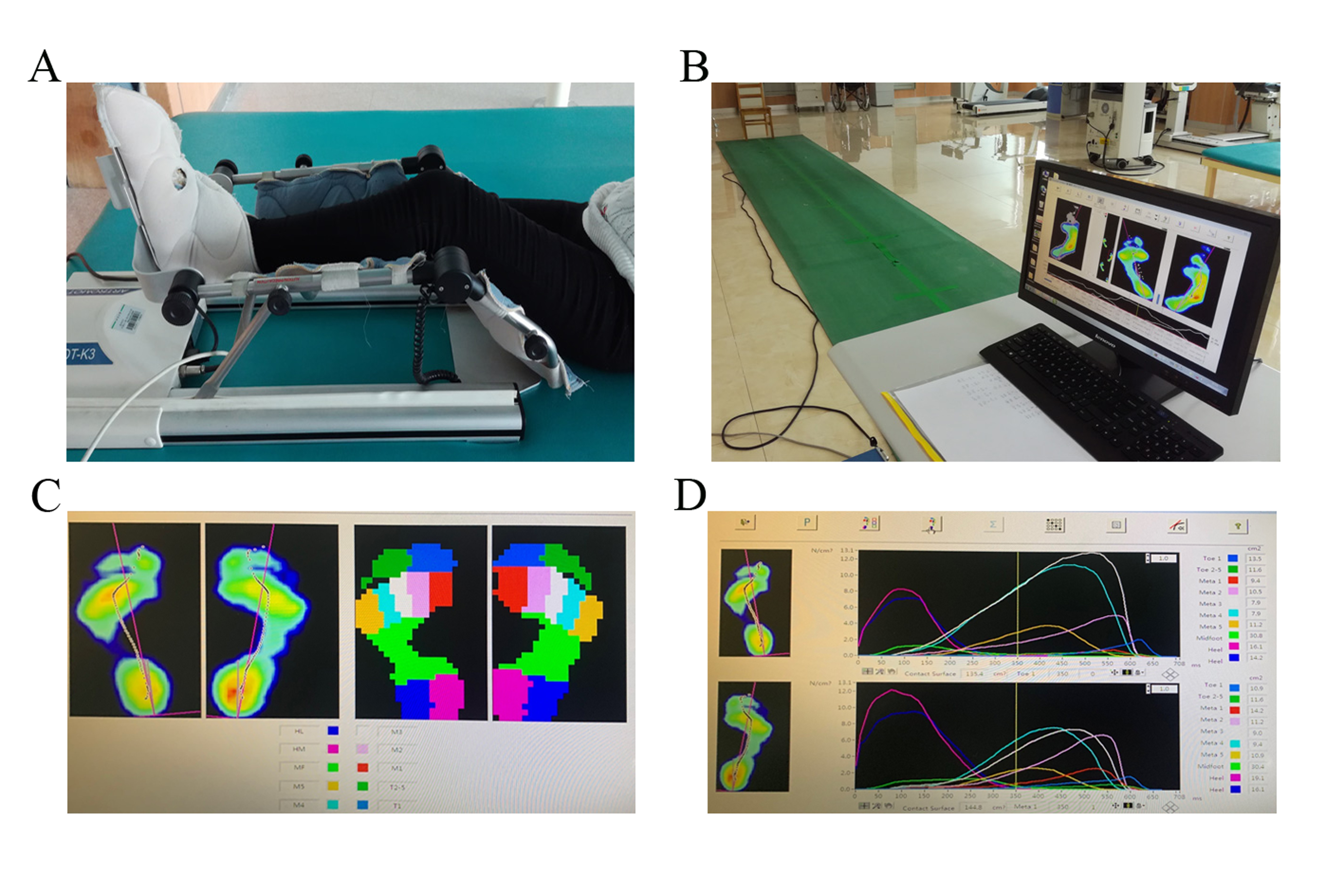

Supplement: Supplementary file 6 [file Image5.JPEG]
